# Pigment fingerprint profile during extractive fermentation with *Monascus anka* GIM 3.592

**DOI:** 10.1186/s12896-017-0366-1

**Published:** 2017-05-25

**Authors:** Kan Shi, Rui Tang, Tao Huang, Lu Wang, Zhenqiang Wu

**Affiliations:** School of Bioscience and Bioengineering, South China University of Technology, Guangzhou, 510006 China

**Keywords:** *Monascus anka*, Extractive fermentation, Pigment profile, New yellow pigments, Bioconversion

## Abstract

**Background:**

Traditional submerged fermentation mainly accumulates intracellular orange pigments with absorption maxima at 470 nm, whereas extractive fermentation of *Monascus spp.* with Triton X-100 can promote the export of intracellular pigments to extracellular broth, mainly obtaining extracellular yellow pigments with absorption maxima at approximately 410 nm. In this study, a strain of *Monascus* (*M. anka* GIM 3.592) that produces high yields of pigments was employed to investigate the differences in pigment fingerprint profiles between submerged and extractive fermentations.

**Results:**

Using extractive fermentation with this high-yield strain, the extracellular pigments exhibited an absorption maximum at 430 nm, not 410 nm, as previously observed. By comparing the pigment fingerprint profiles between submerged and extractive fermentations, extractive fermentation was found to not only export intracellular pigments to the extracellular broth, but also to form four other yellow pigments (**Y1**-**Y4**) that accounted for a large proportion of the extracellular pigments and that were not produced in submerged fermentation. The yields of **Y1**-**Y4** were closely related to the concentration and feeding time point of Triton X-100. **Y1**-**Y4** presented identical UV-Vis spectra with absorption maxima at 430 nm and fluorescence spectra with absorption maxima (emission) at 565 nm. HPLC-MS and the spectral analysis showed that the four pigments (**Y1**-**Y4**) had not been previously reported. The results indicated that these pigments may rely on the bioconversion of orange pigments (rubropunctatin and monascorubrin).

**Conclusions:**

Using extractive fermentation with *M. anka* led to a high yield of extracellular yellow pigments (AU_410 nm_ = 114), and the pigment fingerprint profile significantly differed compared to the results of traditional submerged fermentation. These results provide information and a detailed view of the composition and variation of pigments in extractive fermentation and could also contribute to characterizing pigment metabolism during extractive fermentation.

**Electronic supplementary material:**

The online version of this article (doi:10.1186/s12896-017-0366-1) contains supplementary material, which is available to authorized users.

## Background

*Monascus* pigments are fungal azaphilone metabolites that consist of three groups of pigment components (i.e., yellow, orange and red pigments) that have been used as food colourants in Eastern and Southeastern Asian countries for a long time. The *Monascus* pigment composition mainly consists of two yellow pigments (monascin and ankaflavin), two orange pigments (rubropunctatin and monascorubrin) and two red pigments (rubropunctamine and monascorubramine), all of which are intracellular pigments. By 2012, more than 50 pigments produced by *Monascus. spp* were found and identified [1]. Some of these pigments possess specific bioactivities, such as anti-microbial [2–4], anti-tumour [5–8], anti-oxidative stress [9], anti-obesity [10, 11], anti-inflammation [6, 12, 13] and anti-diabetes [9, 14] properties.

Submerged fermentation (SF) at a low pH can rapidly accumulate a large amount of intracellular orange pigments (rubropunctatin and monascorubrin), independent of the nitrogen sources that are employed [15, 16]. Additionally, SF can avoid generating the mycotoxin, citrinin [17, 18]. In contrast to SF at low pH, extractive fermentation (EF) with Triton X-100 (TX-100) facilitates export of intracellular pigments directly to the fermentation broth, preventing intracellular product inhibition and further degradation of the pigments to improve their yield [19, 20]. In SF, the intracellular pigments mainly consist of orange pigments (rubropunctatin and monascorubrin) with absorption maxima at 470 nm. However, yellow pigments (monascin and ankaflavin) were found to dominate both the intracellular and extracellular pigments, which had the same absorption maxima at 410 nm throughout the EF. This phenomenon was due to the export of intracellular yellow pigments and the subsequent blockage of yellow pigment conversion to orange pigments [21].

In this study, a strain of *Monascus* with a high pigment yield was selected and used for pigment production via EF. The differences in pigment fingerprint profiles between SF and EF were investigated in detail.

## Methods

### Microorganisms and culture

*Monascus anka* (GIM 3.592, Guangdong Culture Collection Centre of Microbiology (GDMCC/GIMCC), China), which was maintained on potato dextrose agar plates and preserved at 4 °C, was used for pigment production. A subculture was carried out at 30 °C for 5 days per month.

The inoculum medium consisted of 20 g of glucose, 10 g of peptone, 3 g of yeast extract, 0.5 g of KCl, 4 g of KH_2_PO_4_, 0.01 g of ZnSO_4_•7H_2_O, and 0.01 g of FeSO_4_•7H_2_O in 1 L of double-distilled water. For inoculum cultivation, a suspension of spores (approximately 3 × 10^6^ spores) was inoculated in 50 mL of the inoculum medium in a 250-mL Erlenmeyer flask. The inoculum was cultivated at 30 °C and 180 rpm for 24 h.

The SF medium consisted of 50 g of glucose, 5 g of (NH_4_)_2_SO_4_, 0.5 g of KCl, 4 g of KH_2_PO_4_, 0.01 g of ZnSO_4_•7H_2_O, 0.5 g of MgSO_4_•7H_2_O, and 0.01 g of FeSO_4_•7H_2_O per litre of double-distilled water. For EF, a certain amount of TX-100 was feed to the fermentation medium at a specific time as indicated. Before inoculation, the pH of all the fermentation media was adjusted to 4.0 with 10% (V/V) hydrochloric acid. For fermentation cultures, an aliquot of the inoculum culture (5 mL) was inoculated into 50 mL of fermentation medium. The fermentation was carried out at 30 °C and 180 rpm for 6.5 days.

For direct extraction of the intracellular pigments using 50 mL of 50 g/L TX-100 aqueous solution (pH was adjusted to 2.0 with 10% (V/V) hydrochloric acid) was made in a 250-mL Erlenmeyer flask, and it was added to 3.0 g aliquots of wet mycelia after 6 days of SF, which were collected under a vacuum through a 0.8 mm mixed cellulose esters membrane and then washed thoroughly with double-distilled water. Meanwhile, as a control, the fermentation supernatant from 6.5 days of SF without mycelia was also collected, and TX-100 was added to a final concentration of 50 g/L (the pH of this solution was approximately 2.0). Then, 50 mL of this solution was added to 3.0 g aliquots of wet mycelia after 6 days of SF in a 250-mL Erlenmeyer flask. The Erlenmeyer flasks were incubated at 30 °C and 180 rpm.

### Analytical methods

#### Pigment, biomass and residual glucose analyses

The fermentation broth in the Erlenmeyer flasks was filtered under vacuum through a 0.8 mm mixed cellulose ester membrane. The collected filtrate (extracellular broth) was normalized to a volume of 50 mL for analysis of extracellular pigments. The mycelia remaining on the membrane were thoroughly washed with double distilled water until the runoff was almost colourless or foamless, and they were then resuspended in 50 mL of acidic aqueous ethanol (70%, v/v, in aqueous hydrochloric acid, pH 2.0) [15, 22] and incubated for 1 h at 30 °C with intermittent shaking. The acidic aqueous ethanol was used to prevent pigment shifting and amination reactions of orange pigments with amino compounds. The suspension was then filtered with pre-weighed filter paper. The filtrate (intracellular extract) was subjected to analysis of the intracellular pigments, and the mycelia remaining on the filter were dried to a constant weight at 60 °C to determine their biomass (dry cell weight, DCW). The comprehensive spectra of the extracellular pigments (i.e., extracellular broth) and intracellular pigments (i.e., intracellular extract) were recorded using a UV/VIS spectrophotometer (Unico, Dayton, OH, USA) from 350 nm to 550 nm at 1 nm intervals. The absorbance units (AU) at specific wavelengths (410, 470 and 510 nm) were used as an index of the extracellular pigment concentration. The residual glucose was quantified using a standard 3,5-dinitrosalicylic acid (DNS) method.

#### Pigment analysis using HPLC-PDAD, HPLC-FLD and TLC

Analytical HPLC was performed with an Alliance e2695 system (Waters, Milford, CT, USA) using a 2998 photodiode array detector (PDAD) (Waters, Milford, CT, USA) and a 2475 multi-wavelength fluorescence detector (FLD) (Waters, Milford, CT, USA). A Sunfire C18 column (250 × 4.6 mm, 5 μm, Waters, Milford, CT, USA) was used and the column temperature was 30 °C. Mobile phase A (aqueous H_3_PO_4_, pH 2.5) and mobile phase B (acetonitrile) were used according to the following gradients at a 1 mL/min flow rate: 0 min, 20% B; 25 min, 80% B; 35 min, 80% B; 36 min, 20% B; and 41 min, 20% B. The PADD was set to 200–600 nm for recording the UV-Vis spectra of the pigments. When recording the pigment profiles and concentrations, the detection wavelength was set to 310 nm. With the FLD, the pigment excitation spectra were recorded from 300 nm to 500 nm at 10 nm intervals using a 565 nm emission wavelength and emission spectra of pigments were recorded from 400 nm to 700 nm at 15 nm intervals using a 331 nm excitation wavelength. Thin-layer chromatography (TLC) analysis was conducted using a Silica gel 60 F_254_ TLC plate (Merck, Germany) with n-hexane/ethyl acetate/petroleum ether (30:17:5) as the developing solvent.

#### Determination of the pigment composition using LC-MS

Liquid chromatography-mass spectrometry (LC-MS) was performed using an HP1100 HPLC system (Agilent, Palo Alto, CA, USA) and a microTOF-QII mass spectrometer (Bruker, Rheinstetten, Germany). The C18 column and chromatographic conditions were the same as the conditions mentioned above, except for mobile phase A (0.1% formic acid in water). The extracellular broth containing high concentrations of TX-100 was processed in advance to remove as much TX-100 as possible before LC-MS analysis. During processing, 6.5-day EF extracellular broth was mixed with isometric diethyl ether to form a Winsor I microemulsion at 4 °C to remove TX-100 [23]. The organic solvent phase (diethyl ether) was collected and diethyl ether was removed by rotary evaporation at 36 °C. The residue was redissolved in an aqueous solution (the pH was adjusted to 2.0 with 10% (V/V) hydrochloric acid), and then the aqueous solution with residue was subjected to microemulsion extraction again. After 3 successive microemulsion extractions, the residue was redissolved in chloroform and a type of post-alkali treated macroporous resin was added to the chloroform. Then, the pigments that were absorbed in the resin were eluted with methanol (0.1% (w/v) hydrochloric acid). The pigments collected after elution were redissolved in chloroform and then submitted to macroporous resin absorption again. In total, the macroporous resin absorption and elution of pigments was successively conducted 4 times to further remove TX-100. The residual pigments were then redissolved in acidic aqueous ethanol (70%, v/v, in aqueous hydrochloric acid, pH 2.0) for LC-MS analysis.

## Results

### Characteristics of pigment metabolism in EF

**Fig. 1:**
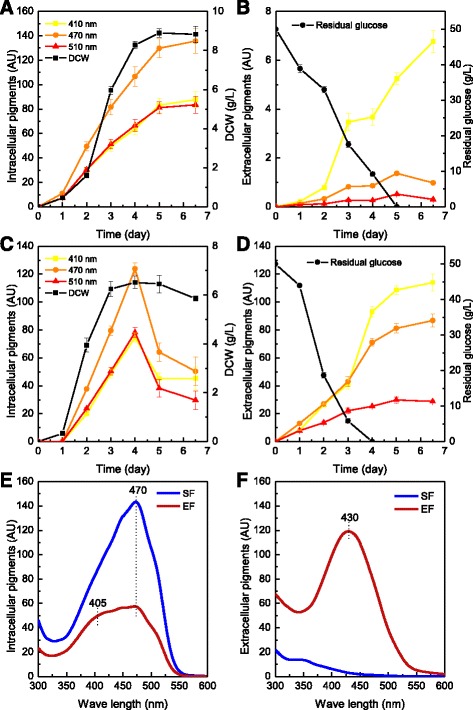
Cell growth, glucose consumption, and intracellular and extracellular pigment production during SF (**a**, **b**) or EF (**c**, **d**). Comprehensive spectra of intracellular (**e**) and extracellular (**f**) pigments after 6.5 days of SF or EF

As shown in Fig. 1a and b, in traditional submerged fermentation without Triton X-100 addition (SF), the pigments mainly accumulated intracellularly, and the AU_470_ can reach 135 after 6.5 days of fermentation. Compared to the production of intracellular pigments, the production of extracellular pigments were extremely low, and the AU_470_ was approximately only 1. On day 6.5, the intracellular AU_470_/extracellular AU_470_ ratio was 138.32, and the intracellular AU_410_/extracellular AU_410_ was 12.98. Both the intracellular and extracellular pigment metabolisms were coupled to cell growth. The logarithmic phase of cell growth began on the 1^st^ day and lasted until nearly the 5^th^ day. The highest biomass (DCW) was 8.9 g/L on the 5^th^ day. Cell growth and pigment production during EF (50 g/L Triton X-100) showed notable differences compared to SF in the beginning (Fig. 1c and d). The highest production of intracellular pigments were obtained on the 4^th^ day when the AU at 470 nm reached 123. However, they decreased sharply after this day. The production of extracellular pigments kept increasing during the entire fermentation, and the final AU at 410 nm reached 114. The extracellular accumulation of pigments was significant after 6.5 days of fermentation. The intracellular AU_470_/extracellular AU_470_ ratio was 0.58 and the intracellular AU_410_/extracellular AU_410_/extracellular AU_410_ ratio was 0.4. The metabolism of the intracellular pigments was coupled to cell growth, but the metabolism of extracellular pigments was not. It was clear that intracellular pigment production lagged for one day. The logarithmic phase of cell growth was shorter and began on the 1^st^ day and stopped on the 3^rd^ day. Compared to SF, EF had much faster glucose consumption. However, cell growth was actually inhibited to some extent, and the highest biomass was only 6.5 g/L.

The comprehensive spectra of the pigments produced during SF and EF showed significant differences (Fig. 1e and f). The extracellular pigment spectra from SF had absorption maxima at approximately 350 nm. However, the pigments from EF showed absorption maxima at 430 nm. The intracellular pigment spectra from SF presented absorption maxima at 470 nm, which indicated that orange pigments were the dominant intracellular pigments. However, in EF, in addition to absorption maxima at 470 nm, the intracellular pigment spectra also had a higher shoulder at 405 nm, which indicated yellow pigments (monascin and ankaflavin) also accounted for a large proportion.

### Pigment fingerprint profiles and the production of four new yellow pigments during EF

**Fig. 2:**
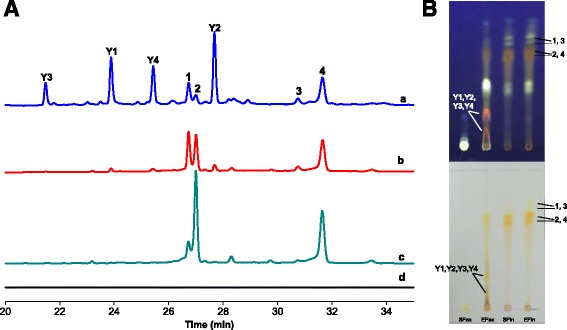
HPLC profiles (**A**) of extracellular pigments of EF (**a**) or SF (**d**), and intracellular pigments of EF (**b**) or SF (**c**) on the 6.5^th^ day. The Y axis for chromatogram c was adjusted appropriately to make the peaks were shown completely. TLC analysis (**B**) of extracellular pigments of SF (SFex) or EF (EFex) and intracellular pigments of SF (SFin) or EF (EFin) on the 6.5^th^ day. The upper image of TLC was taken under ultraviolet light (365 nm). The lower image of TLC was taken under natural light

Because the yield of extracellular water-soluble pigments was extremely low, the pigment analysis was focused on low-polarity pigments. As shown in Fig. 2a, the HPLC pigment fingerprint profile indicated that the intracellular pigments from SF and EF, and the extracellular pigments in SF were mainly low-polarity compounds. It was obvious that compounds **1**, **2**, **3** and **4** were found in the intracellular pigments in SF (Fig. 2A-c) and EF (Fig. 2A-b) and the extracellular pigments in EF (Fig. 2A-a). However, interestingly, besides the four original intracellular pigments (**1**–**4**) produced by *Monascus* strains, EF also produced a large amount of the other four compounds **Y1**, **Y2**, **Y3** and **Y4** (Fig. 2A-a). These four compounds were not produced as extracellular or intracellular pigments in SF.

**Fig. 3:**
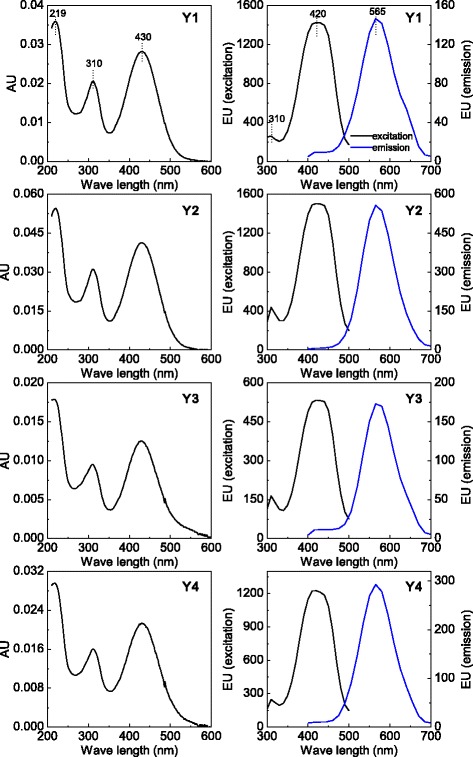
UV-Vis spectra of **Y1**, **Y2**, **Y3** and **Y4** measured by HPLC-PDAD (*left*). Fluorescence excitation and emission spectra of **Y1**, **Y2**, **Y3** and **Y4** measured by HPLC-FLD (*right*)

Compounds **1** and **3** were identified as monascin and ankaflavin (the two major yellow pigments accumulated intracellularly in most *Monascus* strains) by comparison of their UV-Vis spectra and molecular weights with data available in the literature (Table 1) [22, 24, 25]. Compounds **2** and **4** were identified as rubropunctatin and monascorubrin (the two major orange pigments accumulated intracellularly in most *Monascus* strains), respectively, by comparison with data available in the literature (Table 1) [22, 24, 25]. **Y1**-**Y4** had nearly identical UV-Vis spectra with three absorption maxima at approximately 219, 310 and 430 nm (Fig. 3), indicating that they were pigments with a yellow colour, as shown through TLC (Fig. 2b). In addition, as measured by FLD, the results also showed the same fluorescence excitation (Ex) spectra with two absorption maxima at approximately 310 and 420 nm as well as the same fluorescence emission (Em) spectra with absorption maxima at approximately 565 nm (Fig. 3). The fluorescence spectra absorption maxima at 565 nm indicated that the four pigments had orange fluorescence under specific excitation wavelengths; these results were also confirmed by the presence of orange fluorescent bands on the TLC plate under ultraviolet light (365 nm) (Fig. 2b). The molecular weights of **Y1**, **Y2**, **Y3** and **Y4** were 481, 509, 468 and 496, respectively. Thus, based on their UV-Vis spectra, fluorescence spectra and molecular weights, the four compounds have not been described or reported before.

**Table 1:** Chemical characteristics of extracellular *Monascus* pigments during EF

No.	Pigments	Peaks position of spectra (nm)^a^	HRESIMS m/z [M + H]^+b^	Molecular formula^b^
1	Monascin	230, 291, 388	359.1835	C21H26O5
2	Rubropunctatin	214, 247, 286, 471	355.1554	C21H22O5
3	Ankaflavin	230, 293, 388	387.2179	C23H30O5
4	Monascorubrin	214, 274, 285, 471	383.1865	C23H26O5
Y1	Not reported	219, 310, 430	481.1878	--
Y2	Not reported	219, 310, 430	509.2193	--
Y3	Not reported	218, 310, 430	468.2041	--
Y4	Not reported	218, 310, 430	496.2111	--

^a^detected by HPLC-PDAD; ^b^detected by LC-MS

**Fig. 4:**
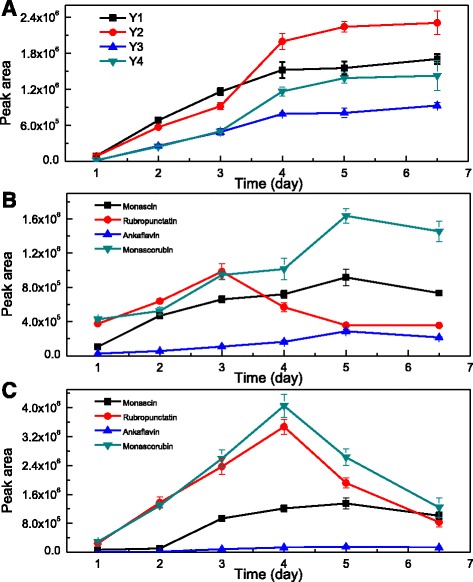
Variation of the peak area of extracellular (**a**, **b**) and intracellular (**c**) pigments during EF measured by HPLC-PDA

The time course for pigment production during EF was recorded based on the pigment peak area variations (Fig. 4). These peak area time courses were mainly used to describe the relative variation of the pigment amount. As shown in Fig. 4a and b, extracellular **Y1**, **Y2**, **Y3** and **Y4**, as well as extracellular monascin and ankaflavin, increased rapidly at the early and middle stage of fermentation. **Y2** and **Y4** kept increasing until the 5^th^ day, **Y1** and **Y3** increased rapidly in the first four days and then increased slightly in the next 2.5 days. At the later stage of fermentation the concentration of intracellular monascin and rubropunctatin was almost 1.4 and 3.6 times higher than the extracellular concentration of monascin and rubropunctatin. During the last 2.5 days, intracellular rubropunctatin and monascorubrin sharply decreased, and their amount on day 6.5 was less than 1/4 of the amount on the 4^th^ day (Fig. 4c). Extracellular monascorubrin increased until the 5^th^ day, while extracellular rubropunctatin increased during the first 3 days and decreased in the following 3.5 days. Both the intracellular and extracellular amounts of monascorubrin were maintained at higher levels than rubropunctatin.

### Effect of the concentration and feeding time point of TX-100 on pigment fingerprint profiles

**Fig. 5:**
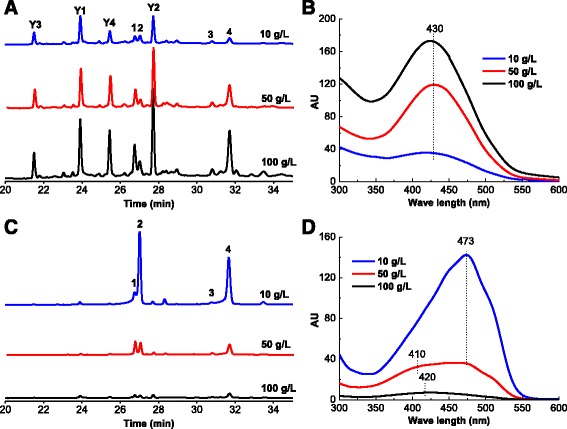
HPLC profiles of extracellular (**a**) and intracellular (**c**) pigments, and comprehensive spectra of extracellular (**b**) and intracellular (**d**) pigments in EF with different TX-100 concentrations (10 g/L, 50 g/L, 100 g/L). The total fermentation time was 6.5 days

Different TX-100 concentrations impact the content of extracellular and intracellular pigments (Fig. 5). The content of extracellular pigments increased significantly with increases of the TX-100 concentration (Fig. 5b, Additional file 1: Figure S1-A and B), and when the TX-100 concentration was 100 g/L, the AU_410_ reached as high as 170. However, **Y1**, **Y2**, **Y3** and **Y4** were detected and accounted for a large proportion of the extracellular pigment fraction despite the different TX-100 concentrations (Fig. 5a), which resulted in the same absorption maxima of the extracellular pigments at approximately 430 nm (Fig. 5b). In contrast to the extracellular pigment content, the intracellular pigment content, especially for rubropunctatin and monascorubrin, decreased significantly with increases of the TX-100 concentration (Fig. 5d and Additional file 1: Figure S1-C). When TX-100 concentration increase from 10 g/L to 100 g/L, the content of intracellular rubropunctatin and monascorubrin (peak area) decreased from 8.02 × 10^6^ and 7.15 × 10^6^, respectively, to 1.61 × 10^5^ and 3.98 × 10^5^, respectively. The intracellular pigments mainly consisted of the two major orange pigments (**2** and **4**) and the two major yellow pigments (**1** and **3**) (Fig. 5c). However, the proportion of each pigment was very different. The intracellular pigments were dominated by the orange pigments (**2** and **4**) when the TX-100 concentration was 10 g/L. When the TX-100 concentration was 50 g/L, the proportion of yellow pigments (**1** and **3**) in the intracellular pigment fraction increased substantially, and the intracellular pigment spectra had absorption maxima at 470 nm with a higher shoulder at approximately 410 nm (Fig. 5d). When the concentration was 100 g/L, the proportion of yellow pigments was higher, and the intracellular pigment spectra had absorption maxima at approximately 420 nm with no absorption maxima at 470 nm (Fig. 5d). The final DCW for EF with 10, 50 and 100 g/L TX-100 was 6.32 ± 0.27, 5.86 ± 0.10, 6.40 ± 0.27 g/L, respectively. Therefore, there was no significant difference among DCW under different concentration of TX-100.

**Fig. 6:**
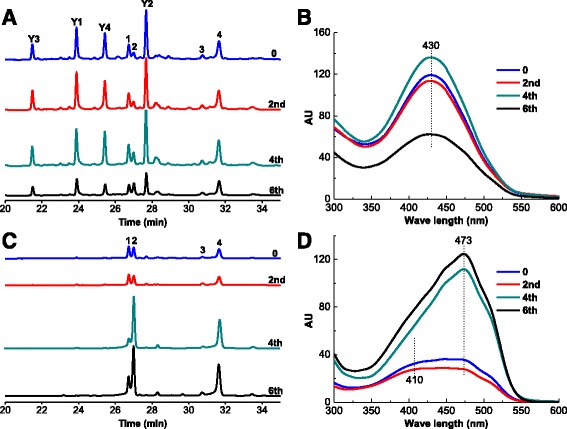
HPLC profiles of extracellular (**a**) and intracellular (**c**) pigments, and comprehensive spectra of extracellular (**b**) and intracellular (**d**) pigments in EF with different TX-100 (50 g/L) feeding time point (the beginning, 2^rd^ day, 4^th^ day or 6^th^ day). The total fermentation time was 6.5 days

For testing the effect of TX-100 feeding time point on the pigment profiles, TX-100 was feed at different fermentation times at a concentration of 50 g/L. The different TX-100 feeding time points had an impact on both the extracellular and intracellular pigment content (Fig. 6 and Additional file 2: Figure S2). Feeding TX-100 on the 6^th^ day gave the highest intracellular pigment content and the lowest extracellular pigment content (Fig. 6b and d). The highest extracellular pigment content was obtained when TX-100 was feed on the 4^th^ day. Feeding TX-100 at an early fermentation stage was shown to give lower amounts of intracellular pigments. The earlier feeding time points would lead to the more thorough exportation of intracellular pigments, especially for the intracellular orange pigments. As shown in Additional file 2: Figure S2, the content of two intracellular orange pigments were around 1 × 10^6^ when TX-100 was feed at the beginning and the 2^nd^ day, whereas the content of two intracellular orange pigments were around 5.5 × 10^6^ when TX-100 was feed at the 4^th^ and 6^th^ day. Whenever TX-100 was feed, the extracellular pigments, which had spectra with the same absorption maxima at 430 nm, were also dominated by **Y1**, **Y2**, **Y3** and **Y4** (Fig. 6a and b). The intracellular pigments were dominated by the orange pigments when TX-100 was feed at a late fermentation stage (4^th^ and 6^th^ day). However, at an early fermentation stage (the beginning and 2^nd^ day), the yellow pigments were a larger proportion of the intracellular pigments (Fig. 6c and d).

### Pigment fingerprint profiles in a TX-100 aqueous solution extraction of mycelia from submerged fermentation

**Fig. 7:**
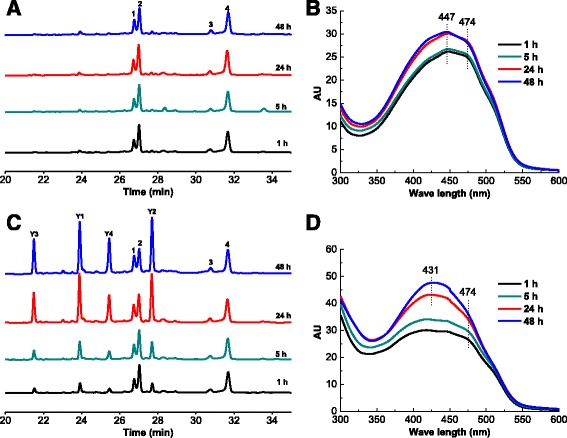
HPLC profiles (**a**) and comprehensive spectra (**b**) of extracellular extract from 6-day SF wet mycelia with a 50 g/L TX-100 aqueous solution (pH 2.0); HPLC profiles (**c**) and comprehensive spectra (**d**) of extracellular extract from 6-day SF wet mycelia with the fermentation supernatant from 6 days of SF without mycelia (adding TX-100 to 50 g/L)

**Y1**, **Y2**, **Y3** and **Y4** were found when TX-100 was feed on the 6^th^ day. Similar to the organic solvent, the TX-100 aqueous solution could also directly extract intracellular pigments from the mycelia. It was mentioned before that SF can only produce the two yellow (**1** and **3**) and two orange pigments (**2** and **4**) (Fig. 2A-c). To determine if direct extraction with TX-100 aqueous solution could yield **Y1**, **Y2**, **Y3** and **Y4**, wet mycelia from 6 days of SF were placed into 50 g/L TX-100 aqueous solution (pH 2.0) and incubated at 30 °C and 180 rpm. Then, the extracellular extract profiles were analysed using HPLC-PDAD and UV-Vis spectroscopy (Fig. 7a and b). Meanwhile, a control group was examined by placing wet mycelia from 6 days of SF into the fermentation supernatant from 6 days of SF without mycelia (adding TX-100 to 50 g/L), and the extracellular extract profiles of this group were shown in Fig. 7c. It was very obvious that a direct extraction with a TX-100 aqueous solution could not produce **Y1**, **Y2**, **Y3** and **Y4** and could only extract the intracellular pigments (**1**, **2**, **3** and **4**) into the extracellular broth. The spectra of the extracellular extract presented an absorption maximum at approximately 447 nm and a high shoulder at approximately 474 nm (Fig. 7b). Additionally, when this direct extracellular extract was mixed with isometric broth from 6 days of SF (without mycelia), the profile of the mixture showed no differences between different incubation times. However, large amounts of **Y1**, **Y2**, **Y3** and **Y4** were produced in the control group, and the amount increased substantially over time (Fig. 7c). These results indicated that the production of **Y1**, **Y2**, **Y3** and **Y4** was closely related to the cellular metabolism of *Monascus*.

## Discussion

In our previous study, EF with TX-100 successfully exported intracellular pigments to the extracellular broth, leading to the accumulation of high amounts of extracellular and intracellular pigments with the same absorption maxima at 410 nm that mainly consisted of yellow pigments (monascin (**1**) and ankaflavin (**3**)) [21]. However, under the same fermentation conditions, SF mainly produces intracellular orange pigments (rubropunctatin (**2**) and monascorubrin (**4**)) with absorption maxima at 470 nm. This interesting phenomenon may be related to the export of intracellular yellow pigments to the extracellular broth by TX-100 and the subsequent prevention of the conversion of yellow pigments into orange pigments. However, in this study, a strain with a high pigment yield (*Monascus anka* GIM 3.592) showed substantial differences with EF. During SF, this strain still mainly accumulated intracellular orange pigments (**2** and **4**) with absorption maxima at 470 nm (Figs. 1e and 2A-c). However, during EF, the intracellular pigments were dominated by neither the yellow pigments nor the orange pigments. Both the yellow and orange pigments were produced at high levels in the intracellular pigment fraction (Fig. 2A-b), resulting in intracellular pigment spectra having an absorption maximum at 470 nm and a high shoulder at 405 nm (Fig. 1e). In addition to the yellow and orange pigments (**1**, **2**, **3** and **4**), EF produced four new yellow pigments with strong orange fluorescence (**Y1**, **Y2**, **Y3** and **Y4**), which all had the same absorption maximum at approximately 430 nm (Fig. 2A-a, B and 3). The four new pigments were a large proportion of the extracellular pigments (Fig. 2A-a), resulting in the comprehensive spectrum of the extracellular pigments having an absorption maximum at approximately 430 nm (Fig. 1f).

The TX-100 aqueous solution can directly extract the intracellular pigments (Fig. 7a). From the 1^st^ to 4^th^ day of EF, the biosynthesis rate of intracellular pigments was higher than the extraction rate of intracellular pigments with TX-100. Therefore, both the extracellular and intracellular pigments accumulated rapidly (Fig. 1c and d). Approaching the end of EF, the biosynthesis rate slowed down or even ceased, but the extraction rate with TX-100 remained high. Therefore, the intracellular pigments decreased sharply in the last 2.5 days. However, as shown in Fig. 7a and c, the accumulation of extracellular pigments during EF cannot be attributed to only the direct extraction of intracellular pigments with TX-100 because direct extraction with TX-100 aqueous solution can only export the intracellular pigments (**1**, **2**, **3** and **4**) but cannot give rise to the four new yellow pigments (**Y1**, **Y2**, **Y3** and **Y4**). Bioconversion among the pigments must exist, resulting in the formation of the four new yellow pigments.

An interesting phenomenon during the last 2.5 days of EF was that the reduction of intracellular rubropunctatin and monascorubrin was not equivalent to the incremental increase of extracellular rubropunctatin and monascorubrin (Fig. 4). The reduction of intracellular monascorubrin was more than 7 times greater than the extracellular monascorubrin increase. Furthermore, both intracellular and extracellular rubropunctatin decreased. Therefore, this remarkable phenomena indicated that the significant missing rubropunctatin and monascorubrin might be the sources of **Y1**, **Y2**, **Y3** and **Y4**. Meanwhile, the accumulation of **Y1**, **Y2**, **Y3** and **Y4** were closely related to the level of the exportation of intracellular orange pigments. The experiments investigating the effect of TX-100 concentration also support this deduction (Fig. 5 and Additional file 1: Figure S1). The different TX-100 concentration had no effect on the final DCW. However, with increases of the TX-100 concentration, intracellular rubropunctatin and monascorubrin decreased significantly (Additional file 1: Figure S1-C), and extracellular **Y1**, **Y2**, **Y3** and **Y4** increased significantly (Additional file 1: Figure S1-A). These results are consistent with a previous study, which found that the exportation of intracellular pigments intensified as TX-100 concentrations increased [26]. Higher TX-100 concentrations helped to export more intracellular pigments and thus left a low level of intracellular pigments. Especially, when TX-100 concentration increase from 10 g/L to 100 g/L, the reduction of intracellular rubropunctatin and monascorubrin (peak area) was approximately 7.85 × 10^6^ and 6.77 × 10^6^, respectively (Additional file 1: Figure S1-C), however, the increase of extracellular rubropunctatin and monascorubrin (peak area) was approximately 4.15 × 10^5^ and 2.66 × 10^5^, respectively (Additional file 1: Figure S1-B). The reduction of intracellular rubropunctatin and monascorubrin was almost than 19 and 26 times greater than the extracellular rubropunctatin and monascorubrin increase when TX-100 concentration increase from 10 g/L to 100 g/L. What is more, when TX-100 concentration increase from 10 g/L to 100 g/L, the reduction of intracellular monascin (1.80 × 10^6^) and ankaflavin (1.96 × 10^5^) was almost equivalent to the incremental increase of extracellular monascin (1.18 × 10^6^) and ankaflavin (2.52 × 10^5^). This results also strongly supported the aforementioned deduction that the missing rubropunctatin and monascorubrin might be the sources of **Y1**, **Y2**, **Y3** and **Y4**.

**Y1**, **Y2**, **Y3** and **Y4** had the same UV-Vis and fluorescence spectra (Fig. 4), indicating that they share a unique chromophore. The difference in the molecular weight between these pigments also indicated that rubropunctatin and monascorubrin might be the sources of **Y1**, **Y2**, **Y3** and **Y4**. The difference in molecular weight between **Y1** and **Y2** and between **Y3** and **Y4** was 28.0315 and 28.0070, respectively (Table 1). The molecular weight difference between rubropunctatin and monascorubrin is 28.0311, which indicated that **Y1** and **Y2** might be formed form the conversion of rubropunctatin and monascorubrin, respectively. The difference of molecular weight between **Y1** and rubropunctatin (126.0324) was almost the same as that between **Y2** and monascorubrin (126.0328). This indicated that **Y1** and **Y2**might be formed by the same modification of rubropunctatin and monascorubrin, respectively. However, the difference of molecular weight between **Y3** and rubropunctatin (113.0487) and between **Y4** and monascorubrin (113.0246). Therefore, **Y3** and **Y4** might be due to the different modification of rubropunctatin and monascorubrin.

## Conclusions

In summary, EF with *Monascus anka* GIM 3.592 not only caused the export of intracellular pigments to the extracellular broth, which led to a high yield of extracellular yellow pigments (AU_410_ = 114), but also altered the composition of pigments. The pigment fingerprint profile from EF showed significant differences from that of traditional SF. In addition to the four original intracellular pigments (**1**-**4**), four new yellow pigments (**Y1**-**Y4**) were detected, and these pigments dominated the extracellular pigment fraction. Furthermore, the results indicated that **Y1**-**Y4** may be formed by bioconversion of orange pigments (rubropunctatin and monascorubrin). These results provide information and a detailed view of the composition and variation of pigments from EF and could also contribute to the elucidation of pigment metabolism during EF.

## Additional files

Additional file 1: Figure S1. Peak area of each pigment in extracellular (A and B) and intracellular (C) pigment fractions in EF with different TX-100 concentrations (10 g/L, 50 g/L, 100 g/L). The total fermentation time was 6.5 days (DOCX 50 kb).

Additional file 2: Figure S2. Peak area of each pigment in extracellular (A and B) and intracellular (C) pigment fractions in EF with different Triton X-100 (50 g/L) feeding time point (the beginning, 2^rd^ day, 4^th^ day or 6^th^ day). The total fermentation time was 6.5 days (DOCX 56 kb).

## Abbreviations

SF: Submerged fermentation
EF: Extractive fermentation
TX-100: Triton X-100
DCW: Dry cell weight
AU: Absorbance units
PDAD: Photodiode array detector
FLD: Fluorescence detector
TLC: Thin-layer chromatography
LC-MS: Liquid chromatography-mass spectrometry
